# An Overview of Factors Associated with Adherence to Lifestyle Modification Programs for Weight Management in Adults

**DOI:** 10.3390/ijerph14080922

**Published:** 2017-08-16

**Authors:** Alice W. Y. Leung, Ruth S. M. Chan, Mandy M. M. Sea, Jean Woo

**Affiliations:** 1 Department of Medicine and Therapeutics, The Chinese University of Hong Kong, Hong Kong, China; ruthchansm@cuhk.edu.hk (R.S.M.C.); jeanwoowong@cuhk.edu.hk (J.W.); 2 Centre for Nutritional Studies, The Chinese University of Hong Kong, Hong Kong, China; mandysea@cuhk.edu.hk

**Keywords:** adherence, lifestyle modification, factors, weight management, adults, obesity

## Abstract

This review aims to provide an overview of the factors associated with adherence reported in existing literature on lifestyle modification programs for weight management among the adult population. An electronic search was performed using PubMed, Medline, PsycINFO and PsycARTICLE to identify studies that examined the factors of adherence to lifestyle modification programs with explicit definition of adherence indicators. We identified 19 studies published between 2004 and 2016. The most commonly used indicator of adherence was attrition, followed by attendance, self-monitoring and dietary adherence. A broad array of factors has been studied but only few studies exploring each factor. Limited evidence suggested older age, higher education, healthier eating and physical activity behaviours, higher stage of change at baseline and higher initial weight loss may predict better adherence. On the other hand, having depression, stress, strong body shape concern, more previous weight loss attempts and being unemployed may predict poor adherence. Inconsistent findings were obtained for self-efficacy, motivation and male gender. This review highlights the need for more rigorous studies to enhance our knowledge on factors related to adherence. Identification of the factors of adherence could provide important implication for program improvement, ultimately improving the effectiveness and the cost-effectiveness of lifestyle modification program.

## 1. Introduction

Overweight and obesity are universal risk factors for non-communicable diseases (NCDs) such as cancer, diabetes and cardiovascular diseases (CVD) [1]. While the prevalence of obesity has been increasing across different age groups, its morbidity and mortality is most frequently manifested in adults [2]. According to the World Health Organization (WHO), it is the fifth leading cause of death in the world, causing at least 2.8 million adult deaths each year [1]. The manifestation of obesity-related morbidity in adult and later life has posed a heavy health care and economic burden on the present and future generations, such that management of obesity in adulthood has become a significant public health concern globally [3]. Nevertheless, efforts to achieve and maintain beneficial weight loss remain a huge challenge for public health professionals [4,5,6].

A review of systematic reviews and meta-analyses on the effectiveness of interventions to reduce or prevent overweight or obesity and improve diet or physical activity published by Stephens et al. suggested that diet and physical activity (PA) were the most common components for various interventions across different settings. Diet-alone interventions appeared to have the greatest effect on weight loss while PA-alone interventions were consistently less effective than diet-alone or multicomponent interventions [7]. Besides, the addition of psychological component further improved the effectiveness of diet and PA interventions [7]. The combination of diet, PA and psychological approaches is commonly referred as lifestyle modification program. Lifestyle interventions on average achieved 7–10% weight loss with the additional benefits of prevention or resolution of obesity-related comorbidities [8,9]. While the results of lifestyle modification programs for weight loss have been promising, generalization of study results is biased by high attrition rates [4,10] and the efficacy on longer term follow up studies is limited by a considerable rebound rate [8,11]. To address these limitations, it is imperative to examine the extent of behavioural changes and explore the potential ways to facilitate and maintain behavioural changes.

The extent of behavioural changes can be explained by the concept of adherence. Adherence was described by the WHO [12] as “the extent to which a person’s behavior-taking medication, following a diet, and/or executing lifestyle changes, corresponds with agreed recommendations from a health care provider”. A synonym commonly used in many publications is “compliance”. Nonetheless, adherence is perceived as more neutral, emphasizing on the self-regulatory actions of an individual while compliance is perceived as paternalistic, emphasizing obedience to instructions. For this reason, adherence is used more often than compliance [13,14]. Nonadherence implies the extent to which a person did not follow recommendations from health care providers, which hampers both external and internal validity or research studies [15]. Attrition, which is an extreme form of non-adherence, is the most commonly reported adherence indicator in lifestyle modification programs as well as other weight loss programs [16,17].

In medical care, better adherence is hypothesized to result in better treatment outcomes [12]. Similarly, consistent positive relationship between adherence to lifestyle modification programs and obesity outcomes was reported in previous studies [18,19,20,21,22,23]. The adherence indicators used in these studies were attendance [18,19,22], self-reported dietary and PA adherence level [18,19,20,21,22], and self-monitoring [22,23]. However, definitions of adherence varied study-by-study, limiting the ability of comparison across study designs.

Measuring adherence helps to indicate the extent to which a program has achieved its specific aims. Consequently, identifying the factors associated with adherence could inform how programs can be improved to facilitate and maintain behavioural changes. To our knowledge, no review on summarizing the factors associated with adherence to lifestyle modification programs has been published to date. A considerable amount of existing literatures has focused on weight loss interventions which are not specific to lifestyle modification programs. Several reviews were identified on this topic. Three of them summarized the factors of weight-related outcomes [24,25,26], which are important clinical indictors but are not sufficient to inform program improvement. The systematical review published by Moroshko et al. [27], summarized predictors of dropout in weight loss interventions including surgical and pharmacological studies. A broad array of correlates and predictors were discussed, including demographic variables, weight/shape factors, dieting/eating behaviour, psychological health, physical health, health behaviours, personality factors and logistics. The authors concluded that a consistent set of predictors could not been identified due to the small number of studies exploring each variable [27]. More recently, Lemstra et al. [28], conducted a meta-analysis of the factors associated with improved adherence to weight loss interventions without any surgical and pharmacological components. Adherence rate was indicated by attendance, percentage of adherence to dietary or PA goal or self-monitoring. Out of the 10 studies that discussed factors affecting adherence, three program characteristics were identified to impact adherence: programs supervising attendance, offering social support, and focusing on diet alone. Other factors that were supported only by few studies include age, income, education, initial weight, poor health, program dissatisfaction, smoking status, and depressed mood [28].

In short, available evidence suggests a wide range of potential factors that may be associated with attrition, attendance, dietary adherence, PA adherence and self-monitoring but existing knowledge is largely drawn from a wide range of weight loss programs that were not specific to lifestyle modification programs. As a comprehensive lifestyle modification program is recognized as the first and most effective option to achieve clinically significant weight loss [4,5,6], there is a need to deepen our understanding of factors associated with adherence to lifestyle modification programs. This paper aims to provide an overview of the factors associated with adherence reported in existing literatures on lifestyle modification programs for weight management among the adult population.

## 2. Method

Literature search was conducted in the following databases: PubMed, Medline, PsycINFO, PsycARTICLES using a combination of the following keywords: diet, physical activity, lifestyle modification, behavioural change, weight loss, weight maintenance, weight control, weight reduction, weight management, factor, determinant, correlate, mediator, predictor, attrition, dropout, adherence, compliance, goal, attendance, self-monitoring (Appendix A). The search was limited to full text English articles in adult population aged 18 and older. There was no time limit on the publication date of articles.

**Figure 1:**
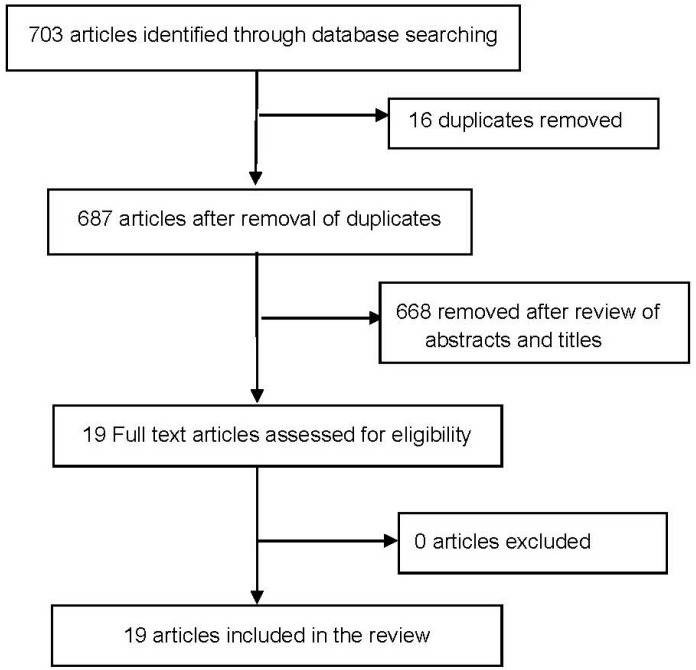
Flow diagram of included studies

This initial search generated 703 articles, of which 16 were removed due to duplication (Figure 1). The titles of 687 articles were initially reviewed for relevance. Full articles of the remaining articles were reviewed according to the following criteria:

1. Lifestyle modification program should not be pharmacological nor surgical and was clearly defined with components of diet, PA and behavioural strategies or theories. The diet component should be based on healthy diet principles and not involve meal replacement, low calorie diet or very-low-calorie diet.
2. Weight management including weight loss and weight maintenance was one of the aim of the studies.
3. Adherence indicators were clearly defined.
4. Studies explored the association between any type of factors and adherence outcomes.
5. Participants were generally healthy without existing chronic diseases, significant psychological comorbidities or any medical condition that limited the ability to perform PA.
6. The main study should be prospective in nature (i.e., cohort studies, controlled trials or quasi-experimental studies).

In total, 19 articles published between 2004 and 2016 were selected based on the inclusion and exclusion criteria. References of these articles were reviewed and no additional articles were found.

## 3. Results

### 3.1. Study and Subject Description

The characteristics of the included studies are presented in Table 1.

#### 3.1.1. Design

All studies are sub-studies of prospective studies with program length ranged from 8 weeks to 6 years. The main studies include nine randomized controlled trials (RCTs) [29,30,31,32,33,34,35,36,37], eight pre/post interventions [38,39,40,41,42,43,44,45] and two longitudinal studies [46,47]. All studies investigated the relationship between baseline factors and adherence outcomes. Only four studies investigated the prospective relationship between factors and adherence outcomes at other time points [36,39,43,45].

#### 3.1.2. Primary Aims of the Studies

The primary aim of most lifestyle modification programs is weight loss [34,35,37,38,39,41,42,43,45,46,47], followed by weight loss and maintenance [32,40,44], prevention of diabetes [31,33], prevention of CVD [30,36] and management of metabolic syndrome [29].

#### 3.1.3. Format and Delivery

The lifestyle modification programs offered were in the form of either groups [30,33,37,38,40,43], individual consultations [31,35,36,42,45,47] or combination of groups and individual consultations [29,32,34,39,41,44,46]. Nearly two third of the studies were community-based [30,31,33,35,37,38,43,44,45,46,47] and the remaining were clinical studies [29,32,36,39,40,41,42]. Among the community-based studies, nearly half were offered through online platforms [34,35,45,46,47]. The intensity of program delivery varied on a program-by-program basis. The frequency of contacts of non-online studies was mostly in weekly basis during weight loss phase and less frequent follow-ups during weight maintenance period. Various interventionists were involved in delivering the programs. Majority of studies were delivered by health professionals (i.e., doctors, dietitians, nutritionists, nurses, physiotherapists, and psychologists) while a few were delivered by trained lay educators.

#### 3.1.4. Lifestyle Modification Components

The basic components of lifestyle modification were identified in most studies but not all studies provided the details of each component. The diet components were mainly based on healthy diet principles, specific recommendations were mentioned in some studies only. For PA, the core recommendation mentioned was to increase PA or encourage regular PA. Specifically, some studies recommended 30–60 min of PA per day. Regarding behavioural components, goal setting and self-monitoring were the two common strategies adopted while cognitive behavioural therapy was the most commonly mentioned therapy.

#### 3.1.5. Subjects

The sample size ranges from 51 to 9599. Subjects were mostly female. Of the 19 studies, seven recruited only female participants [33,35,37,38,44,45,47] and at least half of the subjects in the remaining studies were female. All subjects were overweight or obese with BMI ≥ 25 except one study involved 20% of participants with BMI < 25 [33]. The majority of studies reported mean BMI ≥ 30 [29,30,32,34,35,37,38,39,40,41,42,43,44,45,46]. The mean ages of subjects were at least 40 in over half of all studies.

### 3.2. Adherence Outcomes

A summary of the reported adherence indicators and their corresponding definitions was described in Table 2. The indicators were mostly dichotomous in nature. The most common indicator used was attrition, followed by attendance and self-monitoring. Only one study used dietary adherence as the indicator of adherence.

#### 3.2.1. Attrition

Fourteen studies reported dropout at various time points [29,30,31,32,37,38,39,40,41,42,43,44,46,47]. Nearly all studies recruited participants to join a new program, except Mata et al. who recruited existing participants of two online weight management programs: Brigitte and Weight Watchers. The reported dropout rate from the study was 45.3% and 31.8% respectively [47]. Of the remaining 13 studies, eight studies selected end of program as cutoff point [29,31,38,39,40,42,43,44]; five studies selected cutoff points before the program end [30,32,37,41,46]. The reported % of dropout tends to be higher in clinical studies with more obese participants (30–81.5%) [29,32,40,41] than community-based studies (20.4–42%) [31,37,38,43,44]. High dropout rate was reported in the online program developed by Neve et al. (i.e., 65% at 12 weeks and 70% at 52 weeks) [46]. Among the 14 studies reported dropout rate, 12 examined factors associated with dropout [29,31,32,37,38,39,40,41,42,43,44,46]. One study combined dropout and those who failed to achieve 5% weight loss goal as the dependent variable of analysis [39].

#### 3.2.2. Attendance

Four studies reported attendance as the adherence outcome [30,32,33,47]. Two studies dichotomized attendance as high or low level using two or three sessions as cutoff points (six sessions in total) [30,33]. Around half (48% [33] and 57.4% [30]) of the participants were classified as high attenders. The remaining two studies measured attendance as continuous variables. The mean duration of attendance was 23.15 ± 14.31 weeks in an outpatient weight loss program [40] and 44.1 ± 172 weeks and 38.5 ± 45.3 weeks for two online programs [47]. Among the four studies reported attendance, only three studies investigated predictors of attendance [30,33,47].

#### 3.2.3. Self-Monitoring

Different definitions were found for the three studies used self-monitoring as primary outcomes [34,35,45]. Webb et al. presented self-monitoring as number of weekly journals over a total of 16 weeks. 1 week of completion was defined as completion of food and exercise diaries for at least 5 days per week [45]. Krukowski et al. presented self-monitoring as % of weekly journals over a total of 24 weeks. 1 weekly journal was defined as recording dietary intake, PA, and weight daily for 7 days [34]. The last study by Steinberg et al. assessed self-monitoring via weekly interactive voice response phone calls to record the number of days they achieved their assigned behavioural goals. Adherence was measured in two ways: (i) the proportion of participants who successfully completed calls over the total number of participants expected to complete a call by study week; (ii) the % completion of weekly calls over the 12-month period per participants. Predictors of high completion of self-monitoring (≥80% call completion) were examined in this study [35].

#### 3.2.4. Dietary Adherence

We identified only one study using dietary adherence as indicator of adherence [36]. Dietary adherence was assessed by MEDFICTS dietary assessment tool, with a score of 0–216 [48]. Two binary outcomes were reported: (i) Adherence to Therapeutic Lifestyle Changes diet (TLC) or (ii) Health-Healthy Diet. A score of <40 indicates adherence to TLC (<7% of calories from saturated fat, <30% from total fat and <200 mg of cholesterol per day) while a score of 40–70 indicates adherence to Heart-Healthy Diet (<10% of calories from saturated fat, <30% from total fat and <300 mg of cholesterol per day). Based on these criteria, 36% of participants were non-adherent to TLC diet and 9% were non-adherent to Heart-Healthy diet [36]. Predictors of non-adherence to both diets were examined by univariate and multivariate analyses but only multivariate analysis result of the Heart Healthy model was presented [36].

### 3.3. Factors Associated with Adherence

A broad array of factors has been investigated. A summary of the reported significant factors was presented in Table 2. Majority of studies (10/19) identified predictors through univariate and multivariate analyses [29,32,36,37,38,39,40,41,44,46]. Five reported only multivariate results [30,42,43,45,47] while four conducted univariate analyses only [31,33,34,35]. The factors were further categorized into five groups in descending order of popularity: psychosocial factors (12 studies) [29,30,32,33,36,38,39,40,44,45,46,47], Socio-demographic factors (11 studies) [29,31,34,35,36,37,40,41,42,44,46], behavioural factors (8 studies) [29,31,36,37,38,40,42,46] and physical factors (6 studies) [30,31,36,38,39,43].

#### 3.3.1. Psychosocial Factors

Many psychological factors have been investigated. The most frequently cited were self-efficacy, depression, motivation, stress, body shape concern, quality of life and stage of change.

##### Self-Efficacy

Five studies identified self-efficacy as predictors but the direction of prediction was not consistent [29,30,39,40,47]. Of the five studies, two used validated self-efficacy scales [29,40] while the other three used self-developed questions [30,39,47]. Diet-specific self-efficacy was measured in two studies [29,30]. One clinical study suggested low baseline diet self-efficacy as independent predictor of attrition [29] while one community-based study suggested high baseline diet self-efficacy as predictor of low attendance [30]. Three studies measured general self-efficacy [39,40,47]. One clinical study using both individual and group-based format suggested low baseline self-efficacy as independent predictor of attrition [39] but one group-based clinical study suggested high baseline diet self-efficacy as independent predictor of attrition [40]. The last online community-based study suggested higher baseline self-efficacy as predictor of longer duration spent in the online program [47]. In short, three studies suggested higher self-efficacy but two suggested lower self-efficacy as predictors of higher adherence.

##### Depression

Four studies suggested depression predicted adherence [29,36,38,40]. All studies used validated questionnaires, including Beck Depression Inventory [36,38], Depression scale of Patient Health Questionnaire [40] and Adult Self Report Questionnaire [29]. Two group-based trials suggested depression as a univariate predictor [38,40] and one RCT comparing individual and group-based programs suggested depression as a multivariate predictor [29] of attrition. One individual-based clinical trial suggested depression as univariate predictor of dietary non-adherence [36].

##### Motivation

Four studies reported motivation as predictors of adherence with only half showing positive relationship. Susin et al. measured motivation using the one-item Readiness to Change Ruler in a clinical trial for management of metabolic syndrome. Univariate analysis suggested the dropouts had marginally lower score than the completers [29]. Webber et al. reported higher autonomous motivation, measured by Treatment Self-Regulation Questionnaire, at week 4 predicted higher number of weeks of completion of food and exercise dairies in a 16-week online behavioural weight loss program [45]. On the other hand, two studies found motivation predicted poor adherence. Neve et al. reported the motivation of “1 or more health-related reason for weight loss” as univariate predictor of attrition to a 52-week online weight management program [46]. Toft et al. reported those were “prepared for/minded on exercising more “predicted low attendance to a group-based lifestyle intervention for prevention of CVD compared with those who were not [30].

##### Stress

Three clinical studies found consistent results in the relationship between stress and attrition, where higher stress consistently predicted attrition [29,32,40]. Different validated questionnaires were used to measure stress: Perceived Stress Questionnaire [40], General Health Questionnaire [32] and Stress Symptom Inventory [29].

##### Body Shape Concern

Two community-based studies on women suggested body shape concern predicted attrition [38,44]. One study measured body shape concern through Body Shape Questionnaire [38] and the other through Eating Disorder Examination Questionnaire [44].

##### Stage of Change

Two RCTs aimed for disease prevention identified diet and/or PA-specific stage of change as predictors of adherence. Helitzer et al. measured stage-of-change with seven validated questions corresponding to seven diet and exercise related behaviours. Participants with mean stage-of-change scores corresponding to the action category were more likely to have high attendance in a Diabetes Prevention Program [33]. Aggarwal et al. measured stage-of-change for reducing saturated fat consumption with a simple validated algorithm. Lower stage of change (pre-contemplation, contemplation or preparation) at both baseline and 1-year was found to be an independent predictor of dietary non-adherence in the Family Intervention Trial for Heart Health [36].

##### Quality of Life

Two group-based studies identified quality of life as predictors of attrition. In the community-based study by Teixeira et al., quality of life was measured by Medical Outcome Study Short Form Health Survey SF-36 and obesity-specific IWQOL (Impact of Weight on Quality of Life-lite) [38]. Lower scores in the physical and mental scores of SF-36 and the obesity-specific IQWQOL score both predicted attrition in univariate and multivariate analyses. In the clinical trial by Ahnis et al., physical and mental quality of life were measured by SF-8. Only mental quality of life was identified as a univariate predictor of attrition [40].

Other psychosocial predictors of adherence reported in individual studies were conviction of diet modification [39], mood, sense of coherence, tiredness, positive reframing, anxiety [40], perceived susceptibility of CVD, self-rated care of own health [30], self-esteem, stringent weight outcome evaluation [38], perceived rule complexity [47],subjective complaints, pessimism, avoidant coping, history of mental disorders, alexythimic, perceived mothers overprotecting, maternal care, organization [44] and social support [36].

#### 3.3.2. Socio-Demographic Factors

Age, gender, employment status and education were the four common predictors of adherence.

##### Age

Younger age consistently predicted attrition in four studies using both univariate and multivariate analyses [29,40,42,46] while older age predicted higher % of weekly online self-monitoring journals completed [34] and high completion of self-monitoring calls [35] in univariate analyses. In the study with dietary adherence as outcome, participants who were below 50 years of age had higher odds of being non-adherent to therapeutic diet compared with those aged 50 or above [36].

##### Gender

Being male was a univariate predictor of non-usage attrition of an online program [46] and an independent predictor of attrition of individual counseling sessions in a behavioural weight management program in primary care [41]. In the study with dietary adherence as outcome, male participants had higher odds of being non-adherent to TLC diet or Heart Healthy diet [36]. On the other hand, being male was positively associated with higher % of weekly online self-monitoring journals completed [34].

##### Employment Status

Unemployment was an independent predictor of attrition in two programs consisted of both weight loss and weight maintenance phase [40,44] and univariate predictor of attrition in a clinical study aimed to manage metabolic syndrome [29]. Other than unemployment, having a part-time job was an independent predictor of dropout of group counseling sessions in a clinical study [41].

##### Education

Two studies focused on women reported education as a univariate predictor of adherence. More educated participants were less likely to dropout from a non-dieting group intervention for overweight and obese women [37] and more likely to achieve high completion of self-monitoring calls in an online study [35].

Other socio-demographic predictors of attrition reported in individual studies included having no partners [40], being African American [41], presence of children at home [41], no religion [29] and low socio-economic status [31].

#### 3.3.3. Behavioural Factors

Behavioural predictors were identified for attrition and dietary adherence. The common behavioural factors examined can be grouped into eating or PA behavioural factors and previous weight loss attempt.

##### Eating or PA Behavioural Factors

Four studies consistently reported unhealthy eating behaviours and physical inactivity as predictors of attrition. One online study reported the most specific eating and PA behavioural factors of non-usage attrition of two subscription plans: 12-week and 52-week. Common univariate predictors of non-usage attrition of the two plans were drinking full sugar soft drinks, skipping meals, not eating breakfast, not using low fat products and exercise <2 days per week. The only common independent predictor of non-usage attrition of the two plans was not eating breakfast [46]. In a 16-week group-based weight management program for women, non-completion was positively associated with binge eating, lower baseline carbohydrate and fiber intake and less exercise in univariate analyses. Only baseline carbohydrate intake remained significant in the multivariate model [38]. Eating and PA habit were combined into a subscale score in the Treatment Motivation and Readiness Test (TRE-MORE) questionnaire. Using this questionnaire, Cresci et al. identified lower TRE-MORE lifestyle subscore was an independent predictor of attrition in a clinical individualized weight loss program [42]. Furthermore, binge eating and no PA habit were found to be independent predictors of attrition to a primary prevention program for patients with metabolic syndrome [29]. One study identified only eating behaviour as predictors of attrition. Bradshow et al. investigated lifestyle behavioural factors of non-completion of group non-dieting interventions for overweight women using the Health-Promoting Lifestyle Profile II questionnaire. Lower healthy nutrition-related behaviours subscale scores predicted attrition in both univariate and multivariate analyses [37]. For dietary adherence, the two univariate predictors identified were low PA level and smoking at both baseline and 1-year [36].

##### Previous Weight Loss Attempt

Besides eating or PA behaviour, previous weight loss attempt was a significant predictor in three studies. More previous weight loss attempts predicted dropout in two studies [32,38] while less previous weight loss attempt predicted longer adherence length of an online weight management program [47].

#### 3.3.4. Physical Factors

The major physical factors reported were anthropometric factors. In general, higher baseline weight /BMI/fat [30,31,36,38,39] and less initial weight loss [39,43] predicted poor adherence. Teixeira et al. reported non-completers of a 16-week weight management program were more likely to have higher baseline weight, BMI and body fat [38]. Kong et al. investigated predictors of “loss to follow up or failure to achieve weight loss goal” in an individualized multidisciplinary lifestyle intervention. Higher baseline weight was found to be a univariate predictor while less initial weight loss at 6 weeks was found to be an independent predictor [39]. Higher baseline BMI predicted attrition in an individualized community based lifestyle intervention for prevention of diabetes [31] and low attendance in group-based intervention for prevention of CVD [30]. In a 10-week group based weight loss program, smaller reduction in BMI in the first 2 weeks was the strongest predictor of attrition [43]. In the 9-month clinical trial for heart health, higher BMI and waist circumference measured at both baseline and 1-year predicted dietary non-adherence [36]. Other than anthropometric factors, having glucose intolerance [30,31] and lower aerobic fitness [31] were also reported to be associated with attrition or low attendance.

## 4. Discussion

In this review, we included 19 prospective studies of lifestyle modification programs for weight management in adult population. Similar to other weight management reviews, middle aged women were overrepresented in most studies [16,24,25,26,49].

Adherence was operationalized in four ways: attrition, attendance, self-monitoring and dietary adherence. Most studies explored factors associated with attrition and attendance. Addressing attrition and attendance is important as it helps to identify characteristics of those who would likely succeed and those who need additional support prior joining lifestyle modification programs. Yet, participation in program did not imply the actual lifestyle changes. Self-monitoring is a behavioural strategy commonly used in lifestyle modification programs and dietary adherence measured the dietary changes induced by the program. Therefore, they are better indicators for behavioural change. In this review, only few studies used self-monitoring and dietary adherence as indicators.

Our findings suggested attrition varied among studies, which was comparable to other weight loss interventions [27] and community-based lifestyle modification programs [16]. Attrition was found to be lower in community-based studies with less obese adults than clinical studies. This concurs with our later finding that higher initial weight/BMI as a predictor of attrition. The attrition rate of community-based studies was less than 50%, which concurs with the majority of community-based interventions [50]. However, the attrition rate of clinical studies (30–81.5%) in present review appeared to be higher than previous research involving primary-care physicians (8 to 65%) [49] and patients at low risk of CVD (7–48%) [51].

With the burst of internet and mobile device usage in the past decade, these platforms have a great potential to overcome resource and access barriers incurred in traditional face-to-face settings [52,53]. However, the most effective program will not have public health impact if its actual usage by the target users is low. In our review, only one online study reported non-usage attrition and the rate was relatively high among all 14 studies. This is not surprising as the problem of low actual usage has been a universal challenge in other online studies [53,54,55,56]. A previous systematic review of online prevention programs aimed at lifestyle behaviours suggested a few strategies to increase the use of online programs: sending reminders, incorporating professional support and embedding interventions in existing structures [54].

While attendance was commonly reported as percentage of all sessions completed in existing literatures, studies in our review reported attendance either as dichotomous outcome or the mean duration of program participation. A previous review on weight loss intervention adherence suggested that the average attendance was around 60% [28]. This is comparable to the three group-based studies in our review, where two community-based studies suggested around 50% of all participants attended half or more of the total sessions [30,33] and the remaining clinical study suggested the average duration of treatment around 6 months out of 12 months [40]. One online study reported the average duration of using two commercial online weight loss program were 44.1 weeks and 38.5 weeks [47], which are longer than the optimal 6-month intervention period as suggested by a meta-analysis on the effectiveness of web-based lifestyle modification interventions on weight control [55].

Self-monitoring is the most commonly measured behavioural strategies in behavioural weight loss studies. In our review, all three studies reported self-monitoring were online community-based studies. Of the two studies recorded weekly online food and PA diaries, one did not report the raw self-monitoring data [45], while the other one reported 73% of participants completed at least 1 food and PA diary per week [34]. The third study recorded completion of goals per phone and only around half of the participants completed >80% of the calls [35]. In line with other online weight loss programs [57,58,59], the percentage of self-monitoring was quite low independent of the measurement and analysis used.

Self-monitoring is the key behavioural strategies adopted in lifestyle programs delivered in web-based platforms or mobile devices [52]. Low adherence of self-monitoring might explain the high non-attrition usage of any online behavioural weight loss program. Therefore, addressing the barriers to self-monitoring might also help to alleviate the problem of non-usage attrition. Most frequently reported barriers in earlier studies was related to diet self-monitoring rather than PA self-monitoring [23,60,61]. Recording dietary intake is a cumbersome process and automatic verification of adherence to dietary goals with electronic diet record is challenging due to input error and limitation of functionalities of technology and food database [62]. Developing simple and quick electronic self-monitoring tools might help to improve adherence to self-monitoring as well as non-usage attrition.

Changes in dietary and PA behaviour was the primary goal of lifestyle modification. Yet, we found only one study investigating factors of dietary adherence. None of the studies investigated factors of PA adherence. Dietary adherence was measured using a validated dietary assessment tool based on saturated fat intake. The reported dietary adherence rate was quite high. Comparison with previous studies was impossible due to the variation in assessment of adherence. In previous studies, the commonly reported dietary or PA adherence was operationalized as the percentage of dietary or PA goal. A broad spectrum of adherence rate was reported, ranging from 10 to 87% for diet and 31 to 99% for PA [28]. Dietary adherence was mostly subjectively measured using self-reported scale [63,64,65], food frequency questionnaires [66,67], food diary [22] except one study used an objective measure: Duplicate consecutive DLW assessments [68]. PA adherence was all objectively measured using pedometers [65,69], accelerometer [66] and heart rate monitoring [70].

A broad array of factors was identified and grouped into four categories in our review: psychosocial, sociodemographic, behavioural and physical factors. When taken into account of studies that showed no significant relationship, no consistent predictors of adherence could be identified (Table 3). The number of literatures suggesting significant and non-significant relationship are comparable. This is not surprising as lack of strong evidence was also a major limitation in previous reviews on attrition [27] and weight outcomes management [24,25]. Nevertheless, the directions of relationship for the significant factors identified in our review were largely consistent with previous reviews [24,25,27].

Consistent with other reviews on weight management programs [24,25,26], psychosocial factors were the most widely studied factors. Our findings suggested the presence of depression, stress and strong body shape concern may be predictive of poor adherence to lifestyle modification programs while higher stage of change and better quality of life may be predictive of higher adherence. Similar result for depression and body shape concern have been supported in the review addressing factors of attrition [27] but mixed result was reported in the review on weight managements [24,25,26]. For quality of life, similar result was reported in the review on weight loss for obesity specific quality of life but mixed result was reported for general quality of life [25]. On the other hand, the direction of relationship between self-efficacy and motivation was not consistent in current review. Since both constructs are behavioural specific in nature (diet, PA or weight loss) and different aspects of constructs were reviewed, comparison with other reviews was not possible. In the review addressing pre-treatment psychosocial factors of weight loss, the author suggested mixed findings on relationship between pre-treatment eating or PA self-efficacy and weight loss but consistent relationship between changes in self-efficacy and weight loss [25]. However, higher weight loss specific self-efficacy and motivation were consistently associated with successful weight maintenance [26] and lower attrition rate [27].

Socio-demographic factors were the second most common type of predictors identified in our review but it was the most common type of predictor in the review addressing factors of attrition [27] or adherence [28] among weight loss interventions. Similar to previous reviews, we found younger age and lower education were predictors of poor adherence. With regard to gender, our review suggested being male was a predictor of attrition but also a predictor of higher adherence to self-monitoring. On the contrary, few weight loss studies suggested female as a predictor of attrition [27].

Previous literature provided limited evidence on behavioural and physical factors of adherence and weight management [25,26,28]. We too identified very few studies investigating behavioural and physical factors. Nevertheless, findings on eating and PA behavioural factors in this review generally concur with previous reviews that healthier eating and PA behaviour at baseline may serve as protective factors of poor adherence while unhealthy eating or PA behaviour may be predictive of poor adherence. The most commonly studied eating behaviour was binge eating and all significant findings supported binge eating as predictors of attrition. Other than eating or PA behaviour, less previous weight loss attempts may serve as protective factors of poor adherence. In previous reviews, similar constructs to previous weight loss attempts were investigated. In the review addressing factors of attrition, less previous dieting attempts, was suggested to be a protective factor [27]. Besides, a review on factors of weight maintenance and weight regain suggested weight cycling as predictors for weight regain [26].

For physical factors, only weight-related factors were identified in our review with higher initial weight and lower initial weight loss consistently predicted poor adherence. Other studies have shown consistent result with regard to initial weight loss and attrition [27] or weight maintenance [26] but mixed results with regard to initial weight and attrition [27].

The obesogenic environment has been recognized as the major driving force for the obesity epidemic [71]. Yet, none of the studies investigated environmental factors of adherence. A growing body of literature suggested a consistent relationship between environmental factors (e.g., accessibility to facilities, presence of sidewalks, and aesthetics) and PA behaviour [72,73] while mixed relationship was reported between environmental factors (e.g., accessibility to supermarkets and takeaways) and dietary behaviour in cross-sectional studies among the general population [74,75]. As the effect of environmental influences on adherence to lifestyle modification programs is largely unknown, future studies should examine the environmental factors of adherence to lifestyle modification programs.

## 5. Limitations

Several limitations in this review should be considered. The major limitation is the small number of studies available for many factors identified, particularly when self-monitoring and dietary adherence was the primary outcome. Besides, wide variability in measurement tools, definitions of adherence indicators, intensity of lifestyle modification, assessment time points and sample size also make it difficult to compare across studies. Furthermore, factors were mostly measured at baseline. The temporal aspect of psychosocial and behavioural influence, which often occurs in lifestyle modification program, is largely unknown. Finally, around one-fourth of the studies used only univariate analyses without adjusting for potential confounders which may seriously bias the results.

## 6. Future Research

In light of the limited evidence on factors of adherence to lifestyle modification program, more studies with high methodological rigor is required before any firm conclusions can be drawn. Further research should also focus on behavioural aspects of adherence such as self-monitoring, dietary and PA adherence, which give more practical implications for program improvement. As current literature focuses mainly on weight loss phase, there is a need for more research to investigate the factors of long term adherence to lifestyle modification.

## 7. Conclusions

Research on adherence to lifestyle modification is still at its infancy. We have reviewed a wide range of potential factors related to adherence. Of the 19 studies identified, attrition is the most common indicator used, followed by attendance, self-monitoring and dietary adherence. Factors that may predict better adherence were being in action or maintenance stage of change, older age, higher education, healthier eating and PA behaviour at baseline and more initial weight loss. Factors that may predict poor adherence were depression, stress, strong body shape concern, more previous weight loss attempts and unemployment. Inconsistent findings were found for self-efficacy, motivation and male gender. Despite our conclusions were limited by small number of studies identified for each factor and inconsistent results across studies, our attempt contributes to the synthesis of current knowledge on adherence to lifestyle modification program. More rigorous studies are warranted to enhance our knowledge on factors related to successful lifestyle modification.

## Figures and Tables

**Table 1:** Description of studies exploring factors of adherence to lifestyle modification programs.

Study	Design	Primary Aim	Subjects	Country of Origin	Ethnicity	Setting	Interventionist	Format and Delivery	Dietary Component	PA Component	Behavioural Component	Duration	Assessment of Factors
**Attrition**													
Teixeira et al., 2004 [38]	I	Weight loss	- 158 free-living participants - All F - Age 48 ± 4.5 - BMI 31 ± 3.8	USA	Non-Hispanic or Hispanic white	Community	Intervention team with physical activity, nutrition, psychology, and behaviour modification experts	Group based plus online follow up - 16 weekly group sessions, 150 min each - 25 participants per group	Reducing energy intake to achieve daily energy deficit (less 300–500 kcal/day)	Increase PA to achieve daily energy deficit (less 300–500 kcal/day).	CBT: Goal setting, self-monitoring, self-efficacy enhancement, relapse prevention, contingency management Social support	16 weeks + online contact or no contact for 1 year	Baseline
Kong et al., 2010 [39]	I	Weight loss	- 51 patients with MetS or pre-diabetes - 65% F - Age 50.8 ± 12.0 - BMI 40.5 ± 9.3	Canada	NA	Clinical: Outpatient clinic in an academic hospital	Nurse, endocrinologist and dietitian	Individual based - 1 session every 6 weeks for 1 year - 90 min for first session - 45 min during each follow up session Group based - weekly seminar - walk-in basis	Nutrition goals e.g., (portion sizes, vegetable and whole grain intake, fat content, snacks, caloric beverages) Food labeling Eating out	Long-term objective: 60 min of moderate PA daily	Goal setting Barriers to change Reinforce behaviour Motivation Emotion management Self-esteem	1 year	Baseline + Weight loss data for 6 weeks
Neve et al., 2010 [46]	L	Weight loss	- 9599 participants of a web-based weight loss program 12-week: 6943 52-week: 2656 - 86%F - Age 35.7 ± 9.5 - BMI 32.9 ± 6.7	Australia	NA	Online, Community	Online support from experts	Individual based - 12 or 52-week subscription participants included - Daily calorie goal - Weekly exercise goal - Weekly weigh-ins - Weekly email with educational information Group based - Online discussion forum - Monthly online meeting with other participants	Calorie-controlled and portion controlled diets developed by dietitians.	Step-by-step workout programs Workout videos featuring the Biggest Loser trainers	SCT Goal setting Self-monitoring	12 or 52 weeks	Baseline
Bradshaw et al., 2010 [37]	RCT	Weight loss	- 119 free living individuals with at least 1 CVD factor - All F - Age 25–65 (mean: 46.3) - BMI *Completers:* 34.9 ± 5.4 *Dropouts*: 36.0 ± 6.0	New Zealand	Around 90% New Zealand European	Community	Group 1: Nutritionist and psychotherapist Group 2: Dietitian, psychotherapist and lifestyle activity consultant	Group based - Relaxation response training - Without relaxation response training • 2 h per sessions • 10 initial weekly sessions, then fortnightly and monthly for 8 months	Non-dieting approach (eating regulated by hunger and satiety) Group 1: mindful eating Group 2: low fat diet, food shopping, healthy diet, food variety	Regular PA	Relaxation technique and mindful eating (Group 1 only) Goal setting, self-monitoring, stimulus control (Group 2 only) Body image Enjoyment SCT Cognitive restructuring Coping skills	10 weeks + 8 months (Analysis for first 10 week only)	Baseline
Roumen et al., 2011 [31]	RCT	Prevention of DM	- 147 patients with IGT - 49% F - Age *I:* 55.0 ± 6.5 *C*: 58.8 ± 8.4 - BMI *I:* 29.9 ± 4.2 *C*:29.7 ± 3.4	Netherlands	Caucasian	Community	Dietitian and exercise trainer	Individual based - First session 4–6 weeks after randomization - 1 session every 3 months for 3 years - 1 h per session	Dutch guidelines for a healthy diet	Increase PA at least 30 min a day, 5 days a week Voluntary exercise program Aerobic exercise training Resistance training	Goal setting	3–6 years	Baseline
Ahnis et al., 2012 [40]	I	Weight loss and maintenance	- 164 patients - 84.1% F - Age *Completers:* 47.4 ± 11.0 *Dropouts:* 42.9 ± 11.6 - BMI *Completers:* 39.6 ± 6.5 *Dropouts:* 39.5 ± 6.7	Germany	NA	Clinical-Outpatient clinic	Dietitian, psychologist and physiotherapist	Group based - 2 per week in first 6 months - 1 per week in 6–12 months - 2.5 h each	Balanced diet with reduced fat Reduce intake of food with high glycemic index Reduce 500–800 kcal per day Lectures, controlled dialogue, discussion, group work, theoretical and practical exercises (e.g., cooking)	Movement therapy: equipment-based remedial gymnastics, aqua fitness and medical workout, goal to increase 2–3 h of exercise per week. Muscle relaxation: Jacobson’s progressive muscle relaxation	Psycho-educational and behavioural therapy Self-monitoring Relapse prevention Stimulus Control Behavioural substitution Goal setting	12 months	Baseline
Toth-Capelli et al., 2013 [41]	I	Weight loss	- 461 patients - 84% F - Age 18–55 (38% 40–50) - BMI ≥ 30	USA	60% African American	Clinical-Primary Care	Lifestyle counselor and health educator	Individual based - Periodic sessions, 1 per every 3 months - Occasional phone calls in the first month Group based - Biweekly education group	Food guide pyramid Food labeling Healthy meal planning Supermarket tours Healthy cooking Healthy snacking Dining out Healthier shopping	Incorporate PA into daily life	Goal setting Motivational Interviewing Stage of Change	Periodic, time not specified	Baseline
Cresci et al., 2013 [42]	I	Weight loss	- 266 patients - 73% F - Age 43.2 ± 11.9 - BMI 38.8 ± 6.8	USA	NA	Clinical- Outpatient academic clinic	Endocrinologist (first visit) and dietitian	Individual based - Monthly visit	500 kcal/day reduction diet Individualized diet plan	Endocrinologist provide instruction for PA, details not mentioned	Goal Setting Self-monitoring	6 months, follow up at 1, 2, 4, 6 months	Baseline
Michelini et al., 2014 [32]	RCT	Weight loss and maintenance	146 patients *I*: 73 *C*: 73 74.7% F Age 45 ± 11 BMI 32.3 ± 3.7	Italy	NA	Clinical- Outpatient clinic	Dietitian, physician and psychologist	Group based +Individual based I group (+CBT): 0–6 months: 7 monthly group sessions 90 min each 6–12 months 1 per every 3 months 30 min each Individual visit 12–24 months: 1 per every 6 months 30 min each Individual visit C group: 0–12 months 1 per every 3 months 30 min each Individual visit 12–24 months: 1 per every 6 months 30 min each Individual visit	Both group assigned hypo-caloric diet: • 15% protein; • 55–60% carbohydrate; • 30% lipid; Booklet explaining food groups and portion size	PA for weight maintenance training	CBT: Goal setting, self-monitoring, relapse prevention	24 months (Analysis for 6 months only)	Baseline
Yackobovitch-Gavan et al., 2015 [43]	I	Weight loss	- 587 members of a health care service 90% F Age 46 ± 11 BMI 31.9 ± 5.5	Israel	NA	Community	Dietitian	Group based - 10 weekly sessions - 90 min each - 12 participants each group	Healthy eating habits	Regular PA	Goal setting Coping	10 weeks	Baseline + weight loss data for 10 weeks
Sawamoto et al., 2016 [44]	I	Weight loss and maintenance	- 119 free living individuals - All F Age *Completers:* 47.7 ± 1.2 *Dropouts:* 43.9 ± 2.1 BMI *Completers:* 31.3 ± 0.5 *Dropouts:* 32.0 ± 0.9	Japan	NA	Community	Physician and nutritionist	Group based - 34 weekly sessions + 6 biweekly sessions - 90 min per session - 10 participants per session Individual based - 5 sessions over 44 weeks	Reduction of 500 kcal /day More vegetables Reduction of fatty foods Reduction of sweets	Moderate PA e.g., walk 8000–10,000 steps/day Pedometers provided	CBT: Self-monitoring, stress management	7 months (weight loss) plus 3 months (weight maintenance)	Baseline
Susin et al., 2016 [29]	RCT	Management of MetS	- 127 patients with MetS Group 1: 43 Group 2: 43 Group 3: 41 - 59.1% F - Age 49.6 ± 7.8 - BMI 34.9 ± 3.5	Brazil	87% White	Clinical- Rehabilitation Center in an academic hospital	Physical therapist, psychologist, nutritionist and nurse	Individual based - Group 1: Standard MetS clinical management by nurse - Group 2: • Motivational intervention by psychologist • Weekly nutrition appointments with nutritionist • Performance of exercise monitored by physical therapist Group based - Group 3: • Motivational intervention by psychologist • Weekly group meetings with nurse, physical therapist, and nutritionist	Clinical guideline (Not specified)	Clinical guideline (Not specified)	Motivation Stage of Change	3 months	Baseline
**Attendance**													
Helitzer et al., 2007 [33]	RCT	Prevention of DM	75 free living individuals (I group) All F Age 18–40 BMI > 80% BMI ≥ 25	USA	Indian	Community	Female American Indian health educator	Group-based - 5 monthly class-room sessions - 2–2.5 h each	Increase vegetable intake Reduce dietary fat intake Less sugar and healthy fast food strategies	Regular PA	Social support Relapse prevention -sustain healthy lifestyle behaviours SCT concepts e.g., self-efficacy, expectations, emotional coping	5 months	Baseline
Toft et al., 2007 [30]	RCT	Prevention of CVD	897 free living individuals (I group) 61% F Age 30–60 (58% 40–50) Mean BMI *Low adherence group*: 31.6 ± 0.5 *High adherence group*: 30.8 ± 0.5	Denmark	NA	Community	Nurse and dietitian	Group-based - 6 meetings in 6 months - 15–20 participants per group - 2 h each. - At 1 and 3 years follow up: participants who were still being assessed as high risk underwent the group sessions again	Decreasing saturated fat, substituting saturated fat for unsaturated fat Increasing intake of fruits and vegetables, and fish	Active at least 4 h/week, no intensity requirements (first 6 months) MVPA at least 30 min/day (at 1, 3, 5 years).	Self-perceived health risk Benefit and barriers Self-efficacy Goal setting Motivational Interviewing	6 months	Baseline
Mata et al., 2010 [47]	L	Weight loss	390 participants of two online weight loss programs *B*: 139 *WW*: 251 Al F Age *B*: 39.2 ± 11.6; WW: 33.7 ± 10.34 BMI *B*: 27.9 ± 5.26; *WW*: 29.0 ± 6.00	Germany	NA	Online, Community	NA	Individual based - No common starting point of program - Program length varies and depends on participants’ willingness to pay	*B:* Recipe-based Low calorie diet plan Shopping lists for every meal. *WW:* Point-based system	General recommendations on websites. Weight watchers: Point-based system Brigitte: Individualized exercise plan.	Goal setting: *B*: weight goal; *WW*: time goal; Self-monitoring of diet and PA Problem solving	8 weeks	Baseline
**Self-monitoring**													
Webber et al., 2010 [45]	I	Weight loss	66 free living individuals All F Age 50.1 ± 9.9 BMI 31.1 ± 3.7	USA	86% Caucasian	Online, Community	Nutrition doctoral student	Individual based - 1 Initial face-to-face session by nutrition doctoral student - 16 weekly internet based sessions - 1 group with additional weekly on-line 1-h chat led by nutrition doctoral student - Message board feature - Self-help resources available on the Web	Dietary goals: - low-fat diet (<25% of calories from fat) - low calorie diet of 1200 or 1500 Kcal Overview of energy balance Safe dietary practices Calorie Book	Exercise goal: 30–60 min of MVPA per day Safety recommendations	Goal setting Motivational Interviewing Self-monitoring	16 weeks	Baseline, 4, 8, 12 and 16 weeks
Krukowski et al., 2013 [34]	RCT	Weight loss	161 free living individuals (I group) 93% F Age 46.2 ± 9.8 BMI 35.7 ± 5.7	USA	69% Caucasian	Online, community	Public health practitioner, clinical psychologist and dietitian	Group based - 24 Weekly online group sessions - 12–18 participants per group - 1 h per session Individual based - Weekly feedback on self-monitoring	Calorie-restricted diet ≤25% fat goal	Graded exercise progressed to 200 min/week of MVPA Pedometers provided	Self-monitoring Stimulus control Problem solving Goal setting Relapse prevention Assertiveness training	6 months	Baseline
Steinberg et al., 2014 [35]	RCT	Weight loss	91 free living individuals (I group) - All F Age 35.4 ± 5.5 BMI 30.2 ± 2.5	USA	African American	Online, community (Interactive obesity treatment approach)	Dietitian	Individual based** - Weekly interactive voice response (IVR) calls for self-monitoring of goals - Monthly call with dietitian	≥5 fruit and vegetables/day No fast food No sugar sweetened drinks	Walking 7000 steps/day	Self-monitoring Motivational readiness	12 months	Baseline
**Dietary adherence**													
Aggarwal et al., 2010 [36]	RCT	Prevention of CVD	458 family members of cardiac patients (50% in I group) 66% F Age 49 ± 14 BMI 28 ± 6 (64% with BMI ≥ 25)	USA	65% non-Hispanic White	Clinical-Hospital	Prevention counselor and dietitian (both for I group only)	Individual based - I group: • Stage-matched lifestyle counselling, personalized CVD risk factor assessment • 6 sessions (baseline, 2 weeks,6 weeks, 3, 6 and 9 months) • 30–60 min each - C group: brief, general health message about lifestyle and CVD prevention	Therapeutic Lifestyle Changes (TLC) Diet - Avoid saturated fat, cholesterol, trans fat partially hydrogenated fats - Avoid refined sugars - ≥2 servings fruit/day - ≥3 servings vegetables/day - ≥20 g fiber/day	Moderate PA for at least 30 min per day and 60 min if weight loss was desired	Stage of Change Goal setting Self-efficacy Problem-solving Reinforcing coping skills Reward	9 months	Baseline and 1 year

Key: F: Female I: Pre/post interventions; L: Longitudinal Studies; RCT: Randomized Control Trial; DM: Diabetes Mellitus; CVD: Cardiovascular Diseases; I: Intervention; C: Control; CBT: Cognitive Behavioural Therapy; SCT: Social Cognitive Therapy; IGT: Impaired Glucose Tolerance; MetS: Metabolic Syndrome; B: Brigitte; WW: Weight Watchers; MVPA Moderate to vigorous physical activity; NA: Not Available.

**Table 2:** A summary of the reported adherence outcomes and significant factors of adherence.

Study	Adherence Outcome	Analysis	Significant Factors (*p* < 0.05)	
**Attrition**				
Teixeira et al., 2004 [38]	Dropout at 16 months: 47 (29.7%)	Univariate	+ Psychosocial: (i) Stringent weight outcome evaluation, (ii) Depression, (iii) Body shape concerns + Behavioural: (i) Previous weight loss attempts, (ii) Binge eating + Physical: (i) Initial weight, (iii) Initial BMI, (iii) Initial fat - Psychosocial: (i) Quality of Life (physical, mental and obesity specific) (ii) Self-esteem - Behavioural: (i) Carbohydrate intake, (ii) Fiber intake (iii) Exercise	
		Multivariate	+ Psychosocial: Stringent weight outcome evaluation + Behavioural: Previous weight loss attempts - Psychosocial: Quality of Life (physical, mental and obesity specific) - Behavioural: Carbohydrate intake	
Kong et al., 2010 [39]	Loss to follow up or non-responders (failure to achieve >5% weight loss) at 1 year: 33 (64.7%) ^ Other indicators: Dropout (loss to follow up):15 (30%)	Univariate	+ Physical: Initial weight - Psychosocial: (i) Self-efficacy, (ii) Conviction for diet modification - Physical: % of weight loss at 6 weeks	
		Multivariate	- Psychosocial: Self-efficacy - Physical: % of weight loss at 6 weeks	
Neve et al., 2010 [46]	Non-usage attrition (stopped using the website but active subscription) - 12-week: 4388 (65%) - 52-week: 1429 (70%) ^Other indicators: Dropout for 12-week (<78 days): 238 (3%) Dropout for 52-week (<359 days): 605 (23%)	Univariate	12-week + Socio-demographics: Being male + Behavioural: (i) Eat to ease emotional upset, (ii) Eat to reduce stress, (iii) Drink full sugar soft drinks, (iv) Skipping meals + Physical: Being obese - Socio-demographics: Age - Behavioural: (i) Eat breakfast, (ii) Drink ≥ 6 glasses of water/day, (iii) Use low fat products, (iv) Exercise ≥ 2 days/week 52-week + Behavioural: (i) Fry foods, (ii) Use butter for cooking, (iii) Skipping meals, (iv) Drink full sugar soft drinks, (v) Drink tea or coffee with sugar - Psychosocial: Motivation (≥1 health-related reason for weight loss) - Socio-demographics: Age - Behavioural: (i) Eat breakfast, (ii) Use low fat products, (iii) Exercise ≥ 2 days/week	
		Multivariate	12-week + Behavioural: (i) Eat to ease emotional upset, (ii) Skipping meals - Socio-demographics: Age - Behavioural: (i) Eat breakfast (iii) Exercise ≥ 2 days/week 52-week + Behavioural: Drink tea or coffee with sugar - Behavioural: Eat breakfast	
Bradshaw et al., 2010 [37]	Dropout (<8/10 sessions): 50 (42%)	Univariate	- Socio-demographics: Education - Behavioural: Healthier nutrition behaviours	
		Multivariate	- Behavioural: Healthier nutrition behaviours	
Roumen et al., 2011 [31]	Dropout before 3 years: 32 (21.7%) * [50% from I group] # Result were similar when tested for intervention or control group separately	Univariate	+ Physical: (i) Baseline BMI, (ii) Glucose intolerance - Socio-demographics: Socioeconomic status - Physical: Aerobic fitness	
Ahnis et al., 2012 [40]	Dropout at 12 months: 71 (43.3%) [Breakdown of dropout by 3-month period: 0–3 months: 23 (32.4%) 3–6 months: 17 (23.9%) 6–9 months: 19 (26.8%) 9–12 months: 12 (16.9%)] ^ Other indicators: Attendance (Average duration of treatment): 23.15 ± 4.31 weeks	Univariate	+ Psychosocial: (i) Perceived stress, (ii) Depression, (iii) Anxiety, (iv) Subjective complaints, (v) Pessimism,(vi) Avoidant coping + Socio-demographics: (i) No partners, (ii) Unemployed - Psychosocial: (i) Mood, (ii) Sense of coherence, (iii) Mental quality of life - Socio-demographics: Age	
		Multivariate	+ Psychosocial: (i) Tiredness, (ii) Self-efficacy, (iii) Pessimism, (iv) Positive reframing + Socio-demographics: Unemployed - Psychosocial: Support coping - Socio-demographics: Age	
Toth-Capelli et al., 2013 [41]	**Individual counselling sessions** Dropout (<1 follow up): 327 (70.9%) ^ Other indicators ≥1 follow up visit (1–6 visits): 134 (29.1%)	Univariate	+ Socio-demographics: (i) Being African American, (ii) Being male, (iii) Presence of children at home	
		Multivariate	+ Socio-demographics: (i) Being male, (ii) Presence of children at home	
	**Group class** Drop-out (<1 class): 376 (81.5%) ^ Other indicators ≥1 class: 85 (18.5%)	Univariate	+ Socio-demographics: (i) Being African American or Hispanic, (ii) Part-time employment, (iii) Presence of children at home	
		Multivariate	+ Socio-demographics: Part-time employment	
Cresci et al., 2013 [42]	Drop-out (did not attend all 4 follow ups): 149 (56%)	Univariate	- Socio-demographics: Age - Behavioural: TRE-MORE sub score (current lifestyle habits)	
Michelini et al., 2014 [32]	Overall Dropout: 44 (30%) [Breakdown: - Intervention group (<4 group meetings): 26 (39.7%) - Control group (<2 consecutive visits): 18 (24.7%)]	Univariate	+ Psychosocial: Stress + Behavioural: Previous weight loss attempt	
		Multivariate	+ Psychosocial: Stress	
Yackobovitch-Gavan et al., 2015 [43]	Dropout before week 9: 179 (30.5%)	Multivariate	- Physical: Reduction of BMI in initial stage of the program	
Sawamoto et al., 2016 [44]	Drop-out (Did not complete 7-month weight loss phase): 29 (24.4%)	Univariate	+ Psychosocial: (i) History of mental disorders, (ii) Alexythimic (iii) Strong body shape concern, (iv) Perceived mothers overprotecting + Socio-demographics: Unemployed - Psychosocial: Maternal care	
		Multivariate	+ Psychosocial: (i) Strong body shape concern + Socio-demographics: Unemployed - Psychosocial: (i) Parental bonding-Maternal care, (ii) Perfectionism- Organization score	
Susin et al., 2016 [29]	Drop-out (Did not complete 3-month program): 81 (63.8%)	Univariate	+ Psychosocial: Stress + Socio-demographics: (i) Unemployed, (ii) No religion + Behavioural: Binge eating - Psychosocial: (i) Self-efficacy (diet); (ii) Motivation (readiness to change) - Socio-demographics: Age	
		Multivariate	+ Psychosocial: Isolation and Depression + Socio-demographics: No religion + Behavioural: (i) Binge eating, (ii) No PA habit - Psychosocial: Self-efficacy (diet) - Socio-demographics: Age	
**Attendance**				
Helitzer et al., 2007 [33]	- High attenders (>3 sessions): 36 (48%) - Low attenders (<2 sessions): 39 (52%)	Univariate	+ Psychosocial: Action stage of change (mean of 7 health behaviours)	
Toft et al., 2007 [30]	- High attendance (4–6 sessions): 410 (57.4%) - Low attendance (1–3 sessions): 304 (42.6%) ^ Other indicators Dropout (did not attend any): 183 (20.4%)	Multivariate	+ Psychosocial: (i) High perceived susceptibility of CVD, (ii) Self-rated care of own health + Physical: Screen-detected diabetes or glucose intolerance - Psychosocial: (i) Self-efficacy (diet), (ii) Motivation to increase PA - Physical: Baseline BMI	
Mata et al., 2010 [47]	No. of weeks on current program Brigitte: 44.1 ± 172 weeks Weight watchers: 38.5 ± 45.3 weeks ^ Other indicators Dropout from study Brigitte: 63 (45.3%); Weight watchers: 80 (31.8%)	Multivariate	Brigitte: + Psychosocial: Self-efficacy - Behavioural: Previous weight loss attempts Weight watchers: - Psychosocial: Perceived rule complexity	
**Self-monitoring**				
Webber et al., 2010 [45]	No. of weeks of completion of food and exercise dairies over 16 weeks (≥5 per week) [# mean not reported]	Multivariate	+ Psychosocial: Autonomous motivation at week 4	
Krukowski et al., 2013 [34]	% of weekly journals over 24 weeks (≥1 per week): 73%	Univariate	+ Socio-demographics: (i) Being male, (ii) Age	
Steinberg et al., 2014 [35]	High completion (≥80%) of self-monitoring calls at 12-month: 52% ^ Other indicators Average proportion of participants who completed weekly calls over the no. of expected calls over 12-months: 71.6%	Univariate	+ Socio-demographics: (i) Education, (ii) Age	
**Dietary adherence**				
Aggarwal et al., 2010 [36]	Non-adherent to Therapeutic Lifestyle Changes (TLC) diet (≥40 MEDFITS): 164 (36%) Non-adherent to TLC or Heart Healthy diet (≥70 MEDFITS): 42 (9%) # MEDFICTS: 0–216 points based on 8 food categories	Univariate	Non-adherent to TLC diet + Socio-demographics: Being male + Behavioural: Smoking *^#^ + Physical: (i) BMI *^#^, (ii) WC *^#^ - Psychosocial: Stage of change *^#^ - Socio-demographics: Age - Behavioural: PA *^#^	Non-adherent to TLC or Heart Healthy diet + Psychosocial: Depression ^#^ + Socio-demographics: Being male + Physical: (i) BMI *^#^, (ii) WC *^#^ - Psychosocial: (i) Stage of change *^#^, (ii) Social support * - Behavioural: PA ^#^
		Multivariate		- Psychosocial: Stage of change *^#^

Key: + positive relationship; − negative relationship; PA Physical Activity; BMI Body Mass Index; WC Waist Circumference; * baseline only; ^#^ 1 year; *^#^ baseline and 1-year; ^ Factors associated with other indicators were not reported in the original studies.

**Table 3:** Summary of factors reviewed as predictors of adherence to lifestyle modification programs.

Factors			Relationship	
		Negative	Not Significant	Positive
**Psychosocial**	**Self-efficacy** General Diet PA	[40] [30]	[42] [37,38,43] [29,30,35,38]	[39] [29,47]
	**Depression**	[29,36,38,40]	[35,37,44]	
	**Motivation**	[30,46] (PA)	[30,32,38,42] (Diet)	[29,45]
	**Stress**	[29,32,40]	[35]	
	**Stage of Change**		[39,41]	[33,36]
	**Anxiety**	[40]	[29,37,44]	
	**Social support**		[35] [38] (Diet and PA)	[36]
	**Body shape concerns**	[38,44]		
	**Quality of Life**			[38,40]
	**Self-esteem**	[38]	[44]	
	**Perceived hunger**		[38,40]	
	Others *	Stringent weight outcome evaluation [38]; Subjective complaints pessimism, avoidant coping, tiredness, positive reframing [40]; History of mental disorders, alexythimic, perceived mothers overprotecting [44]; Perceived rule complexity [47].	Mental vulnerability [30]; Weight satisfaction, weight loss expectation [32]; Disinhibition, cognitive restraint [38]; Perfectionism [44]; Intention, planning [47].	Perceived susceptibility of CVD, self-rated care of own health [30]; Conviction for diet modification [39]; Mood, sense of coherence, support coping [40]; Parental bonding- maternal care [44].
**Socio-demographic**	**Age**		[30,31,32,37,38,41,43,44]	[29,34,35,36,40,42,46]
	**Gender (Male)**	[36,41,46]	[29,30,32,42,43]	[34]
	**Employment status** Unemployment	[41] (Part-time job) [29,40,44]	[30,32,35,37]	
	**Education**		[30,32,34,36,41,43,44]	[35,37]
	**Socioeconomic status**		[34,46]	[31];
	**Marital status**	[40] (No partners)	[32,35,36,37,44]	
	**Race**	[41] (Being African American)	[34,36].	
	Others *	Presence of children at home [41]; No religion [29]	Income [35];	
**Behavioural**	**Eating Behaviour** **- *Healthy*** Energy intake Carb ^ intake Fat intake Fiber intake Others *		[31,41,44] [31] [31] [31]	[38] [46] (use low fat products) [38] Healthier nutrition behaviours [37]; Eat breakfast, drink ≥6 glasses of water/day [46].
	**- *Unhealthy***			
	Binge eating	[29,38]	[32,40,44]	
	Others *	Ineffectiveness (Eating Disorder Inventory) [40]; Eat to ease emotional upset, eat to reduce stress, drink full sugar soft drinks, skipping meals, fry foods, use butter for cooking, drink tea or coffee with sugar [46].		
	Self-rated dietary habit		[30]	
	**PA Behaviour**		[30,37,41,44]	
	With PA habit			[36,38,46]
	No PA habit	[29]		
	**Previous weight loss attempts**	[32,38,47]	[42,43]	
	**Smoking habit**	[36,37]	[30,41]	
	**Drinking habit**		[31,41]	
	**Stress management**		[37]	
**Physical**	**Initial weight /BMI /Fat**	[30,31,36,38,39]	[29,32,37,42,43,44]	
	**Initial weight loss**			[39,43]
	**Fitness**		[30] (Physical)	[31] (Aerobic)
	**Glucose intolerance**	[30,31]		
	**Blood pressure**		[31,37]	
	**Cholesterol**		[31]	

Key: * Others: Predictors investigated in 1 study only; ^ Carb: Carbohydrate.

## Appendix A. Example of search strategy used

- Ovid—Ovid MEDLINE(R), PsycAETICLES, PsycINFO
  1. (diet or “physical activity” or exercise* or lifestyle).mp.
  2. (“weight management” or “weight control” or “weight reduction” or “weight loss” or “weight maintenance”). mp.
  3. (factor * or determinant * or correlate * or predictor * or mediator *). mp.
  4. (attrition or dropout or adherence or compliance or goal or attendance or self-monitoring).ti
  5. 1 and 2 and 3 and 4
- Pubmed

(((attrition[Title] OR dropout[Title] OR adherence[Title] OR compliance[Title] OR goal[Title] OR attendance[Title] OR self-monitoring[Title])) AND (diet[Title/Abstract] OR “weight management”[Title/Abstract] OR “weight reduction”[Title/Abstract] OR “weight control”[Title/Abstract] OR “physical activity”[Title/Abstract] OR lifestyle[Title/Abstract] OR “weight loss”[Title/Abstract] OR “weight maintenance”[Title/Abstract])) AND (factor*[Title] OR determinant*[Title] OR mediator* [Title] OR correlate*[Title] OR predictor*[Title])
